# A scoring system and seven factors associated with certification for Japanese long-term care insurance in older people

**DOI:** 10.1007/s00774-025-01606-x

**Published:** 2025-05-28

**Authors:** Keisuke Takahashi, Katsumasa Ideo, Masaru Uragami, Yuko Fukuma, Takehiro Koga, Kazuhiro Yoshiura, Shuken Boku, Naoto Kajitani, Minoru Takebayashi, Takeshi Miyamoto

**Affiliations:** 1 Department of Orthopedic Surgery, Faculty of Life Sciences, Kumamoto University, 1-1- Honjo, Chuo-Ku, Kumamoto, 860-8556 Japan; 2 Department of Physical Medicine and Rehabilitation, Kumamoto University Hospital, Kumamoto, Japan; 3 Department of Frailty Research, Center for Gerontology and Social Science, National Center or Geriatrics and Gerontology, Obu, Japan; 4 Department of Neuropsychiatry, Faculty of Life Sciences, Kumamoto University, Kumamoto, Japan; 5 Center for Metabolic Regulation of Healthy Aging, Faculty of Life Sciences, Kumamoto University, Kumamoto, Japan

**Keywords:** Long-term care insurance (LTCI), Older adults, Scoring system

## Abstract

**Introduction:**

The increase in the older population is a serious concern in developed countries, and how to maintain independence of these individuals is now an urgent issue. Various factors are known to put older people at risk for needing long-term care, but it is not clear to what extent each factor is associated with that need.

**Materials and methods:**

In a cohort of 1577 community-dwelling older persons, we excluded 40 persons whose long-term care insurance certification was unknown and then divided the remaining 1537 into two groups: dependent group (134 persons) certified as requiring assistance or long-term care, and an independent group (1403 persons). We extracted 7 factors and created a scoring system from these factors based on regression coefficients.

**Results:**

Among 92 factors initially evaluated, 7 were significantly associated with the need for assistance or long-term care, namely walking speed, age, grip strength, mobility (EQ5D), ability to use public transportation by oneself (IADL), ability to perform usual activities (EQ5D), and serum albumin levels. Based on these 7, we constructed a scoring system and calculated a cutoff value of 8 points with an area under curve as high as 0.949.

**Conclusion:**

We determined the cutoff value for dependency risk to be 8, but no single factor scored 8 or higher, suggesting that a combination of these factors promotes the need for nursing care in older people.

**Supplementary Information:**

The online version contains supplementary material available at 10.1007/s00774-025-01606-x.

## Introduction

Japan has become a hyper-aged society, and individuals aged 65 and over now account for one-fourth of the total population. This increase in the proportion of the older population continues, and by 2050, it is expected to reach 1/3 of the total population, making measures designed to assist older people an urgent issue.

Since 2000, the Japanese government has introduced a social Long-Term Care Insurance (LTCI) system to support older people with disabilities [1]. Older persons aged 65 and over who are certified and classified based on their physical and cognitive impairments are eligible to receive support from the system (ranging from those in least need of assistance (levels 1 and 2) to those requiring long-term care (levels 1–5) [2]. The need for long-term care exceeds the need for assistance, the latter a term describing a condition requiring moderate assistance in daily living. By contrast, long-term care describes a condition in which individuals need nursing care, and those numbers are currently increasing. In Japan, the number of people certified for long-term care insurance has more than tripled from 2.18 million at the end of April 2000 to 6.80 million at the end of December 2020, and that number is expected to increase further in future. In Japan, the ratio of the older population to the total population continues to increase in combination with decreases in the number of young people and individuals in the working population due to declining birthrates. Accordingly, the total cost of long-term care insurance has increased from 328 billion dollars in 2000 to 811 billion dollars in 2012 and is expected to reach 1821 billion dollars by 2025. Thus, the LTCI system is now in need of comprehensive measures aimed at controlling costs [3, 4].

Various factors reportedly cause older people to require long-term care, including cerebrovascular disorders, dementia, falls, and fractures [5–11]. Comprehensive geriatric assessment (CGA) methodology is used to systematically and comprehensively evaluate not only disease conditions but the status of activities of daily living (ADL), instrumental activities of daily living (IADL) [12], mental and psychological functions, social and economic functions, and quality of life (QOL) in older people [13]. Tools used for this assessment include the Barthel Index for ADL [14], the Mini-Mental State Examination (MMSE) [15] or the Revised Hasegawa's Dementia Scale (HDS-R) for cognitive function, and the Geriatric Depression Scale 15 (GDS15) for emotion and mood [16]. Motivation is also assessed using the Vitality Index [17]. The goal of CGA is to identify older adults at risk for complications and unfavorable outcomes at an early stage, allowing for more appropriate treatment and allocation of multidisciplinary resources. A decline in mobility among older people also drives the decline in an older person's ability to exercise [18]. A decline in motor skills is reportedly directly related to a loss of independence among older people [19]. Quality of life (QOL) can be assessed by the EuroQol 5 Dimension (EQ5D) score. ADL and IADL are used to evaluate independence of older adults [20, 21]. Low serum albumin status is reportedly associated with dependency of older people [22]. However, it is not clear whether combinatorial changes of factors in ADL, IADL, EQ5D, MMSE, GDS15, or physical function or serum albumin levels are associated with the need for long-term care, and to what extent each of these factors contributes to that need.

In the current study, we analyzed 92 factors, among them, age, gender, comorbidities, walking speed, grip strength, serum albumin levels, various individual factors assessed by the Barthel Index, IADL, EQ5D score, GDS15, and the MMSE—all potentially associated with nursing care in older individuals—in a cohort of residents aged 65 years or older living in Arao City, Kumamoto Prefecture, Japan. The purpose of our study was not to deny the usefulness of tools currently used to evaluate independence of older people but rather to define factors and their impact on certification for long-term care needs among older individuals who had actually qualified for social Long-Term Care Insurance. We found that 77 of the 92 factors were significantly associated with the need for care, although their impact differed. We then constructed a scoring system to assess the need for long-term care by weighting these factors in terms of long-term care insurance certification.

## Materials and methods

We enrolled 1577 older people aged 65 years or older living in Arao City, Kumamoto Prefecture, who agreed to participate in this study, the Japan Prospective Studies Collaboration for Aging and Dementia (JPSC-AD), based on a written survey mailed in advance from November 2016 to March 2017 [23]. Interviews were conducted with subjects, and imaging tests, blood tests, and motor function assessments were performed. The survey was conducted at the Arao Municipal Hospital, and subject interviews were conducted by trained physicians, nurses, public health nurses, clinical psychologists, and pharmacists. At that time, we checked whether participants were eligible for assistance or long-term care. Among the 1577, we excluded 40 participants whose assistance or long-term care insurance certification was unknown, and the remaining 1537 remained in the study. Currently, there are seven levels of either assistance or long-term care that require certificates: assistance levels 1 and 2 and long-term care levels 1 (least disabled) to 5 (most disabled), as described in Supplementary Table 1 [24].

Participants were classified as either independent (1403 persons) if they were not certified as requiring assistance or long-term care at the time of the survey or dependent (134 persons) if they were certified as requiring support or nursing care. This study was approved by the Kumamoto University institutional review board, and written consent was obtained from all participants.

### Identification of factors significantly associated with assistance or long-term care

We evaluated 92 factors, all of which were candidate factors associated with the need for care certification, including age, gender, BMI, walking speed, grip power, and cognitive function (Supplementary Table 2). Factors included in the Barthel Index or IADL were used to assess levels of activities of daily living (Supplementary Table 2). Factors in the EQ5D were used to evaluate QOL (Supplementary Table 2), and cognitive function was evaluated using factors in the MMSE (Supplementary Table 2). Finally, emotion and mood were assessed by factors listed in the GDS15 (Supplementary Table 2).

### Extraction of factors correlated with dependency and creation of a scoring system using those factors

First, the objective variable was defined as certification for assistance or long-term care, and logistic regression analysis was used to extract factors that differed significantly between dependent and independent groups (a significant difference was judged to exist when *P* < 0.05). After conducting single regression analysis, BMI, which was comparable in both groups, was excluded from the adjustment factors, and items with significant differences were extracted using age and sex as adjustment factors.

The area under curve (AUC) was then calculated for all extracted factors, and then recalculated by adding factors one by one, starting from the one with the largest AUC, until the AUC reached a plateau, which was then used to create the scoring system. For each factor determined here, a reference was determined and scoring was undertaken. Using that approach, regression coefficients were obtained for each factor, and scores for each were obtained by rounding up decimal points. The total score for each factor was used as the final propensity score. We also evaluated the variance inflation factor (VIF) of the seven factors to analyze collinearity among each factor.

### Statistical analysis

All statistical analyses were performed using EZR [25], which is statistical software that extends the functions of R and R Commander.

## Results

### Basic characteristics of participants

**Fig. 1:**
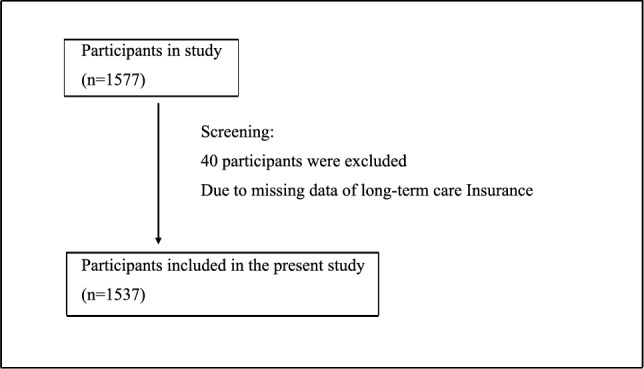
Participants included in the study. We enrolled 1577 community-dwelling older persons. Forty were excluded as their eligibility for assistance or long-term care insurance was unknown at the time of entry. The remaining 1537 participants were included

To identify factors correlated with dependency in older adults, we evaluated a cohort of older adults aged 65 years and older who agreed to participate in the study. To do so, from November 2016 to March 2017, 1577 older adults aged 65 years and older living in Arao City, Kumamoto Prefecture were enrolled, and of those subjects, 40 who had missing data, were excluded, leaving 1537 individuals (569 males and 834 females) as study participants (Fig. 1).

Those subjects were classified into 8 levels of care; independence, 2 levels of support care (assistance levels 1, 2), and 5 levels of nursing care (long-term care levels 1–5). In this system, long-term care ranks higher than assistance, and the higher the number, the greater the level of care. Thus, the highest level of care required was long-term care 5. Participants judged independent numbered 1403 (91.3%), and the remaining 134 (8.7%) fell into either assistance (63 participants, 4.1%) or long-term care (71 participants, 4.6%) categories. Of these 134 persons, 37 were in assistance 1, 26 in assistance 2, 32 in long-term care 1, 22 in long-term care 2, 9 in long-term care 3, 7 in long-term care 4, and 1 in long-term care 5 (Table 1).

**Table 1:** Basic levels of care of participants in the two study groups

	Independent	Requiring assistance (1,2) or long-term care (1–5)
	(*n* = 1403)	(*n* = 134)
Nursing care level, *n* (%)		
Independent	1403 (100%)	0 (0%)
Assistance1	0 (0%)	37 (27.6%)
Assistance2	0 (0%)	26 (19.4%)
Long-term care 1	0 (0%)	32 (23.9%)
Long-term care 2	0 (0%)	22 (16.4%)
Long-term care 3	0 (0%)	9 (6.71%)
Long-term care 4	0 (0%)	7 (5.22%)
Long-term care 5	0 (0%)	1 (0.75%)

We then divided study subjects into two overall groups: independent (corresponding to the independent group described above) and dependent, including all subjects who required assistance or long-term care. The age of dependent individuals (83.55 ± 7.01 years) was significantly higher than the ages of independent individuals (73.66 ± 6.15 years) (*p* = 6.66e^−39^) (Table 2). In terms of gender, 37 of a total of 606 men (6.1%) and 97 of 931 women (10.4%) were dependent, with a significantly higher proportion of women being dependent (Table 2). On the other hand, there was no significant difference in BMI between dependent and independent groups (Table 2).

**Table 2:** Basic characteristics of participants in the two study groups

	Independent	Requiring assistance (1,2) or long-term care (1–5)	Odds ratio (95%Cl)	*P* value
	(*n* = 1403)	(*n* = 134)		
Age (years, mean ± SD)	73.66 ± 6.15	83.55 ± 7.01	1.24 (1.2–1.28)	6.66e^−39^
Gender, *n* (%)			1.79 (1.21–2.65)	3.77e^−3^
Male	569 (40.6%)	37 (27.6%)		
Female	834 (59.4%)	97 (72.4%)		
BMI (kg/m^2^), mean ± SD	23.48 ± 3.23	22.96 ± 3.81	0.95 (0.90–1.01)	0.082

Odds ratios and *P* values were determined by univariate analysis of logistic regression analysis

### Factors significantly associated with certification of long-term care insurance

Since there were significant differences in age and gender between dependent and independent groups, we analyzed 92 factors, such as age, sex, comorbidities, lifestyle habits, physical functions (grip power and walking speed), serum albumin levels (Table 3) and factors included in scores, such as EQ5D (Supplementary Table 3), Barthel index (Supplementary Table 4), IADL (Supplementary Table 5), GDS15 (Supplementary Table 6) and MMSE (Supplementary Table 7), using logistic regression analysis with age and gender as adjustment factors in single regression analysis. Seventy-seven out of 92 factors analyzed differed significantly between dependent and independent groups (Table 3 and Supplementary Tables 3–7).

**Table 3:** Odds ratios of each factor evaluated for assistance or long-term care (LTC) certification in participants

Factors	Odds ratio (95% Cl)	*P* value	Adjusted Odds ratio for sex and age (95% Cl)	P value
Age	1.240 (1.200–1.260)	6.66e^−39^		
Sex	1.790 (1.210–2.650)	3.77e^−3^		
Stroke	4.930 (3.090–7.850)	2.02e^−11^	4.600 (2.620–8.080)	1.12e^−7^
Heart failure	8.410 (3.780–18.70)	1.82e^−7^	7.550 (2.870–19.90)	4.12e^−5^
Head injury	4.460 (2.170–9.180)	4.89e^−5^	4.160 (1.730–10.00)	1.46e^−3^
Parkinson’s disease	15.60 (4.890–50.00)	3.51e^−6^	29.40 (7.560–114.0)	1.04e^−6^
Cranial nerve-related diseases	5.690 (2.780–11.70)	2.01e^−6^	5.260 (2.160–12.80)	2.65e^−4^
Hypertension	2.790 (1.840–4.230)	1.31e^−6^	2.160 (1.360–3.430)	1.10e^−3^
Drinking	0.861 (0.631–1.180)	0.345	1.600 (1.100–2.320)	1.33e^−2^
Smoking	0.830 (0.653–1.060)	0.131	1.450 (1.020–2.050)	3.95e^−2^
EQ5D^*1^	1.06e^−4^ (2.43e^−5^–4.59e^−4^)	2.90e^−34^	7.02e^−4^ (1.52e^−4^–3.24e^−3^)	1.27e^−20^
Barthel Index*^2^	0.772 (0.734–0.812)	5.22e^−24^	0.812 (0.768–0.857)	8.08e^−14^
IADL*^3^	0.560 (0.514–0.609)	6.07e^−41^	0.609 (0.556–0.666)	3.73e^−27^
Exercise habits	0.522 (0.363–0.750)	4.40e^−4^	0.598 (0.392–0.913)	1.72e^−2^
Grip	0.835 (0.808–0.863)	1.73e^−26^	0.837 (0.804–0.873)	3.64e^−17^
Walking speed	2.180 (1.850–2.570)	1.52e^−20^	1.690 (1.460–1.950)	1.48e^−12^
Albumin	0.062 (0.035–0.110)	7.99e^−22^	0.195 (0.105–0.362)	2.42e^−7^
Dementia	4.860 (3.770–6.280)	5.36e^−34^	2.790 (2.070–3.770)	1.53e^−11^
GDS15 score*^4^	1.260 (1.190–1.340)	1.58e^−14^	1.220 (1.140–1.310)	3.26e^−8^
MMSE score*^5^	0.767 (0.733–0.802)	3.06e^−31^	0.832 (0.792–0.873)	1.51e^−13^

*1, *2, *3, *4, *5: Factors evaluated for each score are described in Supplementary Tables 3–7

We then calculated the area under curve (AUC) for each factor that differed significantly between independent and dependent groups (Supplementary Table 8). Then, AUCs were recalculated by adding one factor at a time, starting with the factor with the largest AUC, which in this case was walking speed, with an AUC of 0.875. However, when we recalculated the AUC using two factors, namely walking speed and age, which had the second largest AUC, the AUC was 0.896. The same process was repeated 6 more times, and on the 8th round—once we added the question, “Are you able to prepare meals by yourself? (IADL)”—the AUC reached a plateau (Supplementary Table 9). However, we later determined that the question “Are you able to prepare meals by yourself? (IADL)” is influenced by gender. Therefore, we deleted it from our final scoring system when we selected 7 factors associated with dependence (Tables 4 and 5).

**Table 4:** AUC (Area under the ROC curve) for each factor evaluated for assistance or LTC certification

Factors	AUC (95% CI)
Walking speed	0.875 (0.842–0.907)
Age	0.849 (0.814–0.884)
Grip	0.811 (0.771–0.852)
Mobility (able to walk, or bedridden) (EQ5D)	0.805 (0.767–0.844)
Able to use public transportation by oneself (IADL)	0.794 (0.752–0.835)
Able to perform usual activities (EQ5D)	0.769 (0.727–0.812)
Albumin levels	0.737 (0.688–0.787)

*LTC* Long-term care

**Table 5:** AUC analysis of factors significantly associated with Dependency

Factors	AUC (95% CI)
Walking speed	0.875 (0.842–0.907)
Age	0.896 (0.865–0.926)
Grip	0.898 (0.864–0.931)
Mobility (able to walk, or bedridden) (EQ5D)	0.916 (0.885–0.947)
Able to use public transportation by oneself (IADL)	0.939 (0.918–0.961)
Able to perform usual activities (EQ5D)	0.942 (0.922–0.963)
Albumin levels	0.943 (0.922–0.963)

The AUC was calculated by increasing factors one by one, starting with the factor showing the highest AUC value, as indicated in Table 4

We then selected a reference for each category and conducted logistic regression analysis to obtain regression coefficients. Those references were walking speed, 1.0 m/s or more; age, 65–74 years; grip strength, ≥ 28 kg (men) and ≥ 18 kg (women); mobility (EQ5D), "can walk"; ability to use public transportation by oneself, "able" (IADL); ability to perform usual activities (EQ5D), "able"; and serum albumin levels, < 4 g/dl. All factors differed significantly from the reference, *P* < 0.001 (Table 6).

**Table 6:** Odds ratios of 7 factors evaluated for assistance or LTC certification from each reference value

Factors	Odds ratio (95% Cl)	*P* value
Walking speed, m/s	2.18 (1.85–2.57)	1.52e^−20^
(vs ≥ 1)		
0.8 < < 1	9.16 (5.37–15.6)	4.34e^−16^
≤ 0.8	39.1 (21.0–72.8)	8.08e^−31^
Age, years	1.24 (1.20–1.28)	6.66e^−39^
(vs 65–74)		
75–84	5.96 (3.34–10.7)	1.65e^−9^
85–89	24.4 (12.6–47.2)	2.15e^−21^
90–	168 (69.9–404)	2.25e^−30^
Grip strength	2.80 (2.33–3.35)	7.62e^−29^
(vs men ≥ 28, women ≥ 18, kg)		
Men < 28, Women < 18	8.48 (5.81–12.4)	1.64e^−28^
Mobility (EQ5D)	17.8 (11.8–26.8)	5.80e^−43^
(vs able to walk)		
Bedridden	18.3 (12.1–27.6)	7.86e^−43^
Able to use public transportation by oneself (IADL)	0.016 (0.01–0.03)	1.09e^−62^
(vs able)		
Not able	63.2 (38.9–103)	1.09e^−62^
Able to perform usual activities (EQ5D)*^1^	18.4 (12.3–27.4)	2.31e^−46^
(vs able)		
Not able	21.6 (14.4–32.4)	
Albumin levels	0.062 (0.035–0.110)	7.99e^−22^
(vs≧4 g/dl)		
< 4 g/dl	7.40 (4.86–11.3)	1.17e^−20^

*LTC* Long-term care

*1 work, study, family and leisure activities

### Establishment of a scoring system to evaluate dependency risk

Beta coefficients were used as regression coefficients, and decimal points were carried forward to obtain risk scores for each item listed below.

For walking speed, 1 m/s or more scored 0, > 0.8 to < 1 m/s scored 3, and 0.8 m/s or less scored 4. For age, 65–74 years scored 0, 75–84 years scored 2, 85–89 years scored 4, and 90 years or more scored 6. For grip strength, 28 kg or more for men or 18 kg or more for women scored 0, and < 28 kg for men or < 18 kg for women scored 3. For mobility (EQ5D), bedridden scored 3. Relevant to the ability to use public transportation by oneself (IADL), "not able" scored 5, and for ability to perform usual activities (EQ5D), "not able" scored 4. For albumin levels, < 4 g/dl scored 3. Risk scoring was then performed by summing the scores of each category above. The total score ranged from 0 to 28 (Table 7).

**Table 7:** Scores associated with nursing care level

Predictors	Regression coefficient	score
Walking speed, m/s		
0.8 < < 1	2.2151	3
≤ 0.8	3.6653	4
Age, years		
75–84	1.7856	2
85–89	3.1957	4
90–	5.1241	6
Grip		
Men < 28, Women < 18	2.1378	3
Mobility (EQ5D)		
Bedridden	2.9042	3
Able to use public transportation by oneself (IADL)		
Not able	4.1466	5
Able to perform usual activities (EQ5D)		
Not able	3.0718	4
Albumin levels		
< 4 g/dl	2.0008	3

**Fig. 2:**
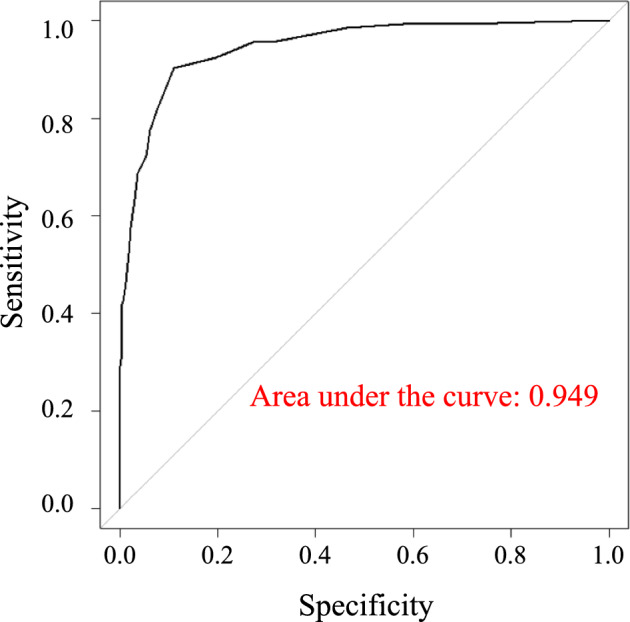
Receiver operating characteristic curve for factors significantly associated with assistance or long-term care certification. We calculated the area under the curve (AUC) for factors significantly associated with assistance or long-term care certification, namely, Age, Barthel index score, IADL score, EQ5D score, walking speed, grip strength, and MMSE score, and determined it to be 0.946

**Fig. 3:**
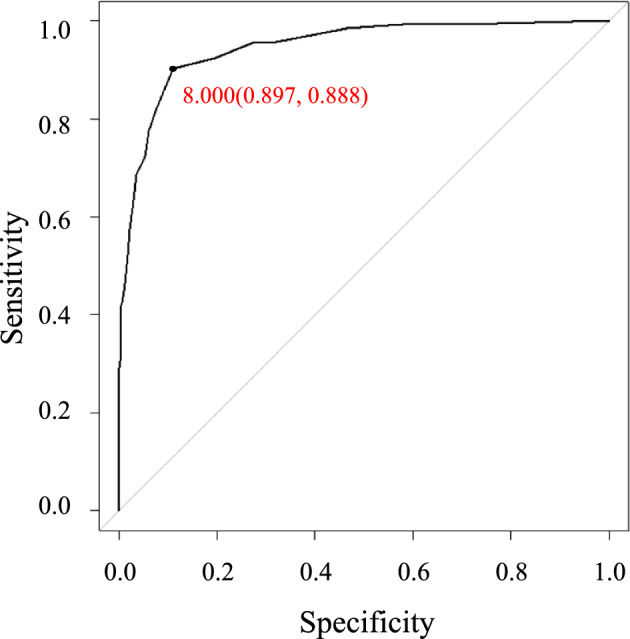
Cutoff score for factors significantly associated with assistance or long-term care certification. We examined the cutoff score for factors significantly associated with assistance or long-term care certification by evaluating the diagnostic accuracy of quantitative tests using ROC curves. The cutoff score calculated based on the sum of sensitivity and specificity was determined to be 8 with a sensitivity of 0.903, specificity of 0.890, and an AUC of 0.946 (95% CI 0.926–0.966)

**Fig. 4:**
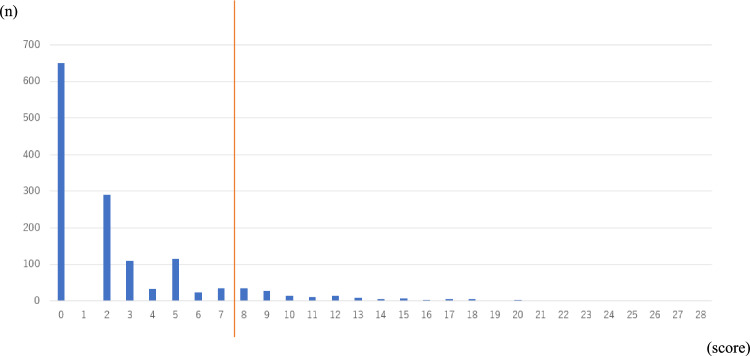
Total score distributions of participants in the independent group. Histogram analysis showing distribution of total scores of 1,403 individuals in the independent group. The number of individuals scoring 8 or higher was 155 out of 1403

**Fig. 5:**
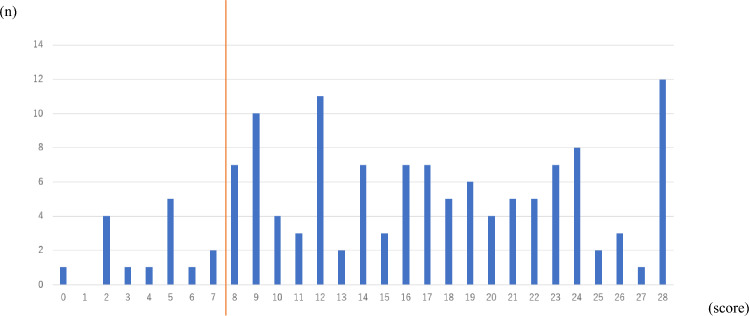
Total score distributions of participants in the dependent group. Histogram analysis showing distribution of total scores of 134 individuals in the dependent group. The number of individuals scoring 8 or higher was 121 out of 134

Finally, to assess cutoff scores, we evaluated the predictive performance of the risk score by calculating the area under the ROC curve (AROC) and examined sensitivity, specificity, positive predictive value, and negative predictive value. The cutoff score was also determined using the ROC curve to evaluate accuracy of the quantitative test for diagnosis. We determined that 8 points (sensitivity: 0.897, specificity: 0.888) to be the maximum sum of sensitivity and specificity, respectively. The AUC was 0.949 (95%, CI 0.930–0.967) (Figs. 2 and 3). Among 1403 participants in the Independent group, 145 (10.3%) scored 8 or higher, compared to 119 of 134) participants (88.9%) in the dependent group (Figs. 4 and 5).

## Discussion

Societal aging society is now a global concern as countries experience an increase in the older population and a decrease in the relative proportion of young people [26]. Thus, maintaining the independence of older people is now a critical issue worldwide [27]. Older individuals lose independence due to various diseases and physical disability, but there have been few systematic studies of combinatorial factors, including physical functions, underlying this loss or their relative importance. To address this need, we conducted a cohort study of 1577 community-dwelling older persons aged 65 years or older and identified 77 factors significantly associated with the need for assistance or long-term care. We calculated the AUC for all 77 and then recalculated AUCs by adding candidate factors one by one, starting with the top AUC values, to identify 7 factors with a plateau AUC as previously described [28]. When we evaluated only these 7 factors, the AUC was equivalent to that seen after evaluating all 77 factors. The 7 factors were slow walking speed, advanced age, decreased grip strength, being bedridden (EQ5D), unable to use public transportation by oneself (IADL), unable to perform usual activities (EQ5D), and low serum albumin status, specifically, < 4 g/dl. In this study, in addition to walking speed and grip strength, each item in EQ5D and IADL was evaluated in relation to the certification for long-term care insurance as an explanatory variable, and thus, it might be possible that these factors are not independent each other. Meanwhile, when the variance inflation factor (VIF) of the seven factors identified in this study was evaluated, the VIF among all items was less than 2 (Supplementary Tables 12), suggesting no collinearity. We then constructed a scoring system with cutoff values for each factor and regression coefficients. In this system, we calculated the cutoff value associated with the need for support and care to be 8. Each of the 7 factors showed a maximum score of 6 in subjects aged 90 years or older, indicating that reaching or exceeding the cutoff value of 8 requires evaluating combinations of these factors in order to determine the need for support and care.

Older people with dementia are tended to be certified for long-term care insurance. Indeed, we demonstrated that MMSE total score and various items assessing MMSE were significantly associated with the certification for long-term care insurance. However, the AUC analysis did not include cognitive impairment as one of top factors among the items associated with certification for long-term care insurance. This is likely that the seven factors identified in this study are more significantly associated with the certification for long-term care insurance than cognitive impairment. In the current study, by comparing groups that had been certified for long-term care with those that had not, we identified factors that were significantly associated with the group that had been certified for long-term care. The findings may be useful in constructing preventive methods for the certification of older persons as needing long-term care. Meanwhile, individualized analysis and personalized care are important to prevent dependency of the older people.

Except for age, a factor significantly associated with the need for support and care identified here was “Not able to use public transportation by oneself (IADL)”, which was scored as 5, the second highest score in our scoring system. All IADL factors were significantly associated with the need for assistance or long-term care; moreover, the inability to travel alone by public transportation was most associated with need for support and care (Supplementary Table 3) [21, 29]. In EQ5D analysis, the item related to self-care was most related to the need for support and care (Supplementary Table 4) [30]. For each item of the Barthel index, “Toilet use” was most associated with the need for assistance or long-term care (Supplementary Table 5) [31]. All of these conditions predict a decline or impairment in activities of daily living and were extracted as items associated with the need for assistance or long-term care and were scored according to their weighting, which we believe indicates our scoring system's validity. Similarly, all MMSE factors were significantly associated with the need for assistance or long-term care (Supplementary Table 6). Thus, maintaining cognitive function is considered essential for older people to maintain independence. A decline in cognitive function was previously reported to be associated loss of independence in older people, an observation that also indicates the validity of this scoring system [32–34]. For each of the GDS15 items, a positive response to “Do you often feel helpless?” was most significantly associated with need for assistance or long-term care (Supplementary Table 7). Meanwhile, low albumin status was previously known to indicate low nutritional status and a poor general condition overall [22], and indeed, it was identified as a factor significantly associated with the need for assistance or long-term care.

By contrast, low walking speed [9, 21, 35–37] and decreased grip strength [9, 38] are both diagnostic criteria for sarcopenia, and the fact that scores were assigned to these factors suggests that decreased physical function and muscle strength are major factors in loss of independence among older people. In particular, decreased walking speed had the largest AUC, suggesting that loss of this function is directly related to loss of independence in older people [39, 40].

Older individuals have diverse needs and their overall condition cannot be assessed based on a single factor. Thus, we evaluated individuals' condition by combining age, gender, comorbidities, walking speed, grip strength, serum albumin levels, individual items within the Barthel index, IADL, EQ5D score, GDS15, and the MMSE. The Barthel index and IADL assessments directly assess independence of older people based on each item in that tool. In our study, we calculated the odds ratio to indicate the impact of each item of the Barthel index and IADL on long-term care insurance certification (Supplementary Tables 3 and 5). The impacts of ADL and IADL on long-term care insurance certification might be improved by a more comprehensive evaluation including physical functions. Furthermore, we demonstrated that among 1403 participants in the Independent group, 145 (10.3%) scored 8 or higher, compared to 119 of 134 (88.9%) in the dependent group (Figs. 4 and 5). Thus, it will be necessary to conduct a longitudinal study to verify whether the 145 persons in the independent group who scored 8 or higher will become dependent in future.

In developing our scoring system, we evaluated AUC scores for each factor on long-term care insurance certification and found that physical factors like walking speed and grip strength ranked first and third, respectively. Furthermore, osteoporotic fragility fractures, particularly, hip fractures, are reportedly risks for loss of independence in older individuals [41]. Osteoporosis and sarcopenia are known to be significantly associated with each other in the very old [42], and lower walking speed and decreased grip strength are both criteria for diagnosing sarcopenia [43].

We analyzed various comorbidities, namely stroke, heart failure, head injury, Parkinson's disease, cranial nerve-related diseases, hypertension, and dementia, as shown in Table 2, but other comorbidities, namely diabetes and chronic kidney disease, also reportedly contribute to the need for support and care [44]. Taken together, we conclude that including assessment for osteoporosis, sarcopenia, fragility fracture and other comorbidities will increase the utility of our system and enable prediction of a future need for nursing care. Further studies are necessary to confirm this possibility. Also, since this is a cross-sectional study, further longitudinal study is needed.

Study limitations include the fact that it is cross-sectional, and although the identified factors are associated with assistance or long-term care, their causal relationship to that need remains unclear. There is a possibility that care-needing situations were not properly evaluated in some cases. It is also not clear whether study results can be extrapolated globally since we enrolled subjects from only one geographic region. In an attempt to be exhaustive, the CGA includes numerous evaluation items and is generally considered complicated and time-consuming. Thus, simplified evaluation charts, such as Total Kihon checklist, CGA7, and Dr. SUPERMAN, have been developed [45–47]. Our new scoring system includes serum albumin status, which enables more accurate assessment of older people's condition but requires blood sampling, which can be time-consuming. Thus, we provided an alternate scoring system without albumin measurement (Supplementary Table 10 and 11). In that system, we determined 8 points (sensitivity: 0.911, specificity: 0.881) to be the maximum sum of sensitivity and specificity, respectively, and the AUC was 0.949 (95% CI, 0.930–0.967) (Supplementary Figs. 1 and 2). In the independent and dependent groups, 125 (8.91%) and 118 (88.1%), respectively, scored 8 or higher (Supplementary Figs. 3 and 4). Finally, the system is not suitable for large-scale studies, such as those utilizing questionnaires for evaluation. Although we have tried to narrow down relevant items based on AUC, further simplification of the system is also important.

In conclusion, analysis of real-world data revealed the impact of factors on certification for long-term care among older people who had obtained that certification. To assess this impact, we used a scoring system that weighted factors used to calculate Barthel index, IADL, MMSE, EQ5D and GDS15 scores, and then determined a cutoff value useful to identify individuals entitled to certification for long-term care. We also determined the extent to which each factor was associated with the need for long-term care. We feel this knowledge will be helpful in understanding causes of conditions associated the need for long-term care certification and potentially prevent or delay conditions that underlie these needs in older people not yet certified. Identifying factors characteristic relevant to individuals actually certificated for long-term care insurance should help us devise interventions to prevent or delay certification for long-term care insurance and potentially decrease insurance cost. Identifying people with lower scores could help suggest interventions to maintain cognitive or physical function, such as improving walking speed, in order to help older people maintain their quality of life and prevent or delay the need for additional support [48].

## Supplementary Information

Below is the link to the electronic supplementary material.

Supplementary file1 (PPTX 174 KB)

Supplementary file2 (DOCX 66 KB)
