# Supplementary material for: A scoring system and seven factors associated with certification for Japanese long-term care insurance in older people
Source: J Bone Miner Metab. 2025 May 28;43(4):419–29. doi: 10.1007/s00774-025-01606-x (PMC12279562; doi:10.1007/s00774-025-01606-x)
Supplement: Supplementary file 1 — Supplementary file1 (PPTX 174 KB) [file 774_2025_1606_MOESM1_ESM.pptx]

## Slide 1
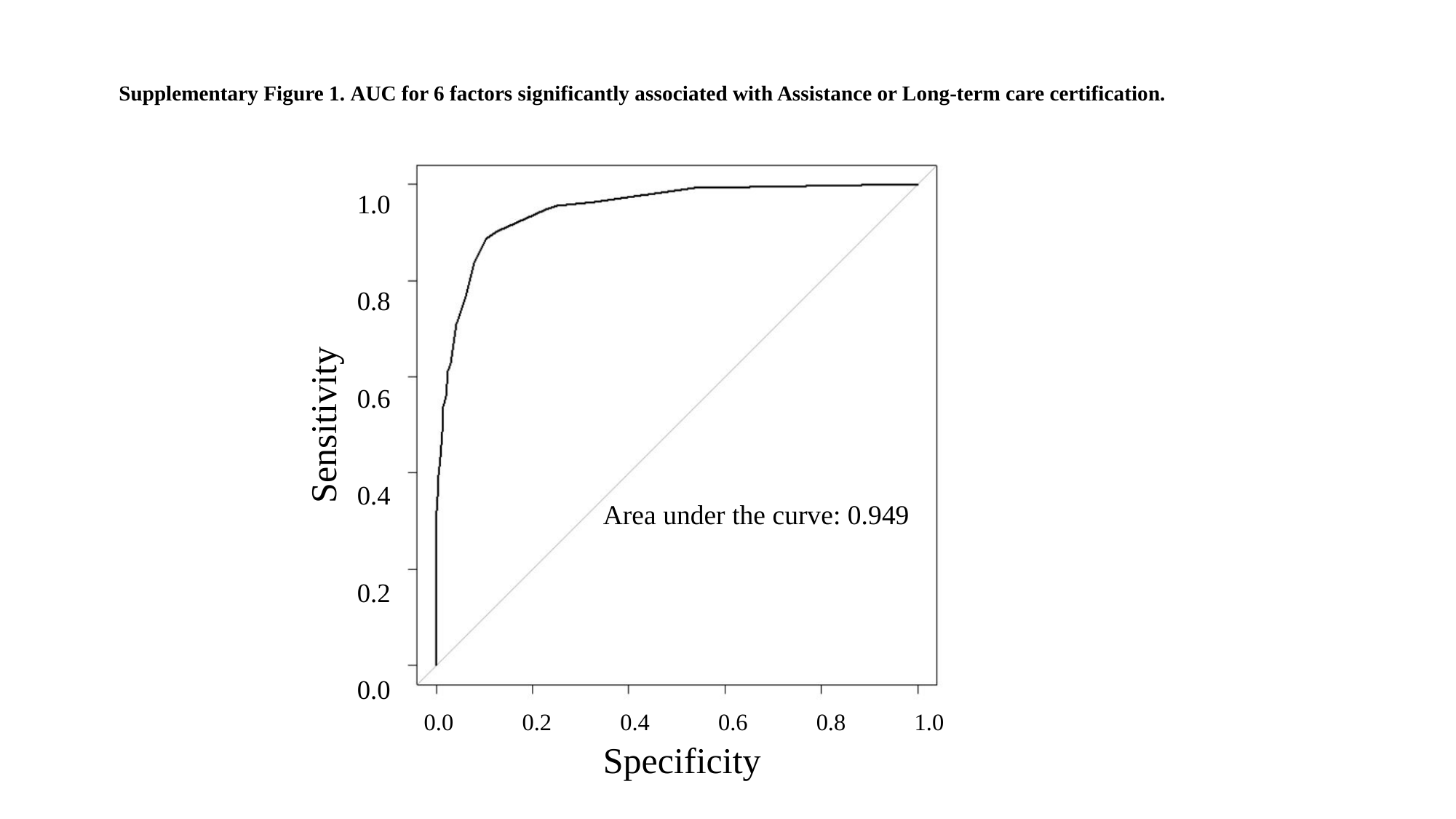

Supplementary Figure 1. AUC for 6 factors significantly associated with Assistance or Long-term care certification.
1.0
0.8
0.6
0.4
0.2
0.0
Sensitivity
Area under the curve: 0.949
0.0　　 0.2　　 0.4　　 0.6　　 0.8　　 1.0
Specificity

## Slide 2
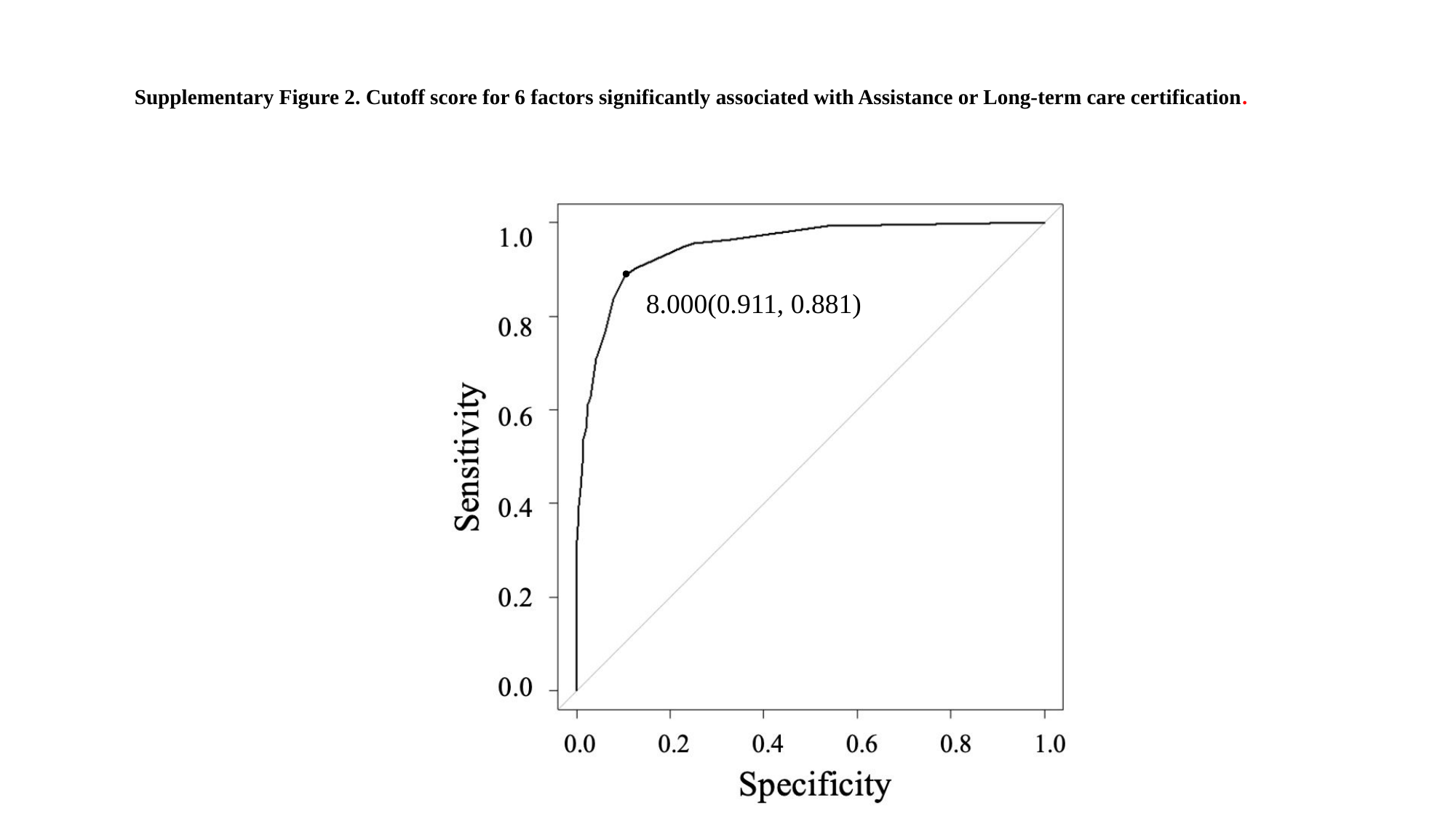

Supplementary Figure 2. Cutoff score for 6 factors significantly associated with Assistance or Long-term care certification.
8.000(0.911, 0.881)

## Slide 3
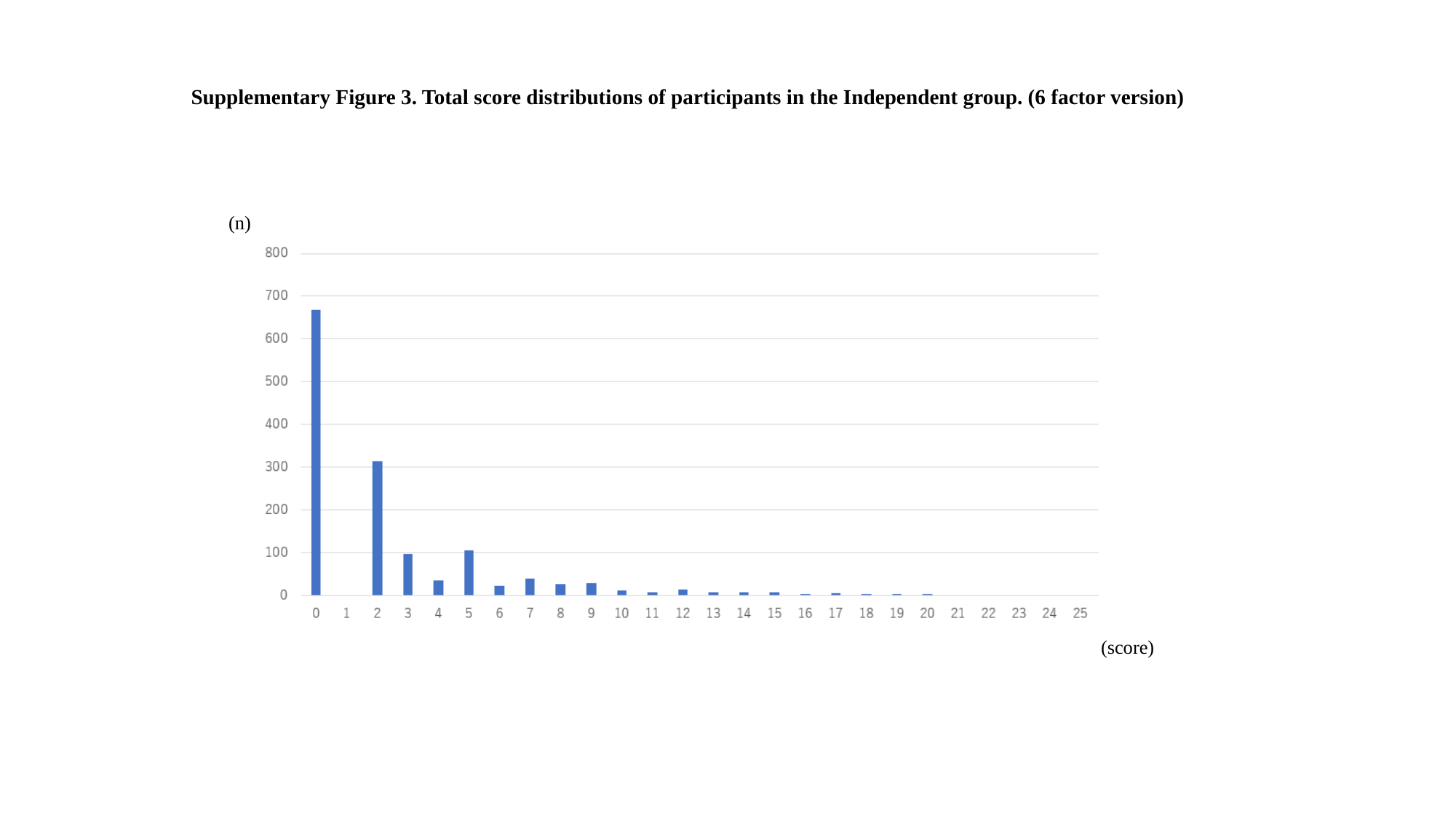

Supplementary Figure 3. Total score distributions of participants in the Independent group. (6 factor version)
(n)
(score)

## Slide 4
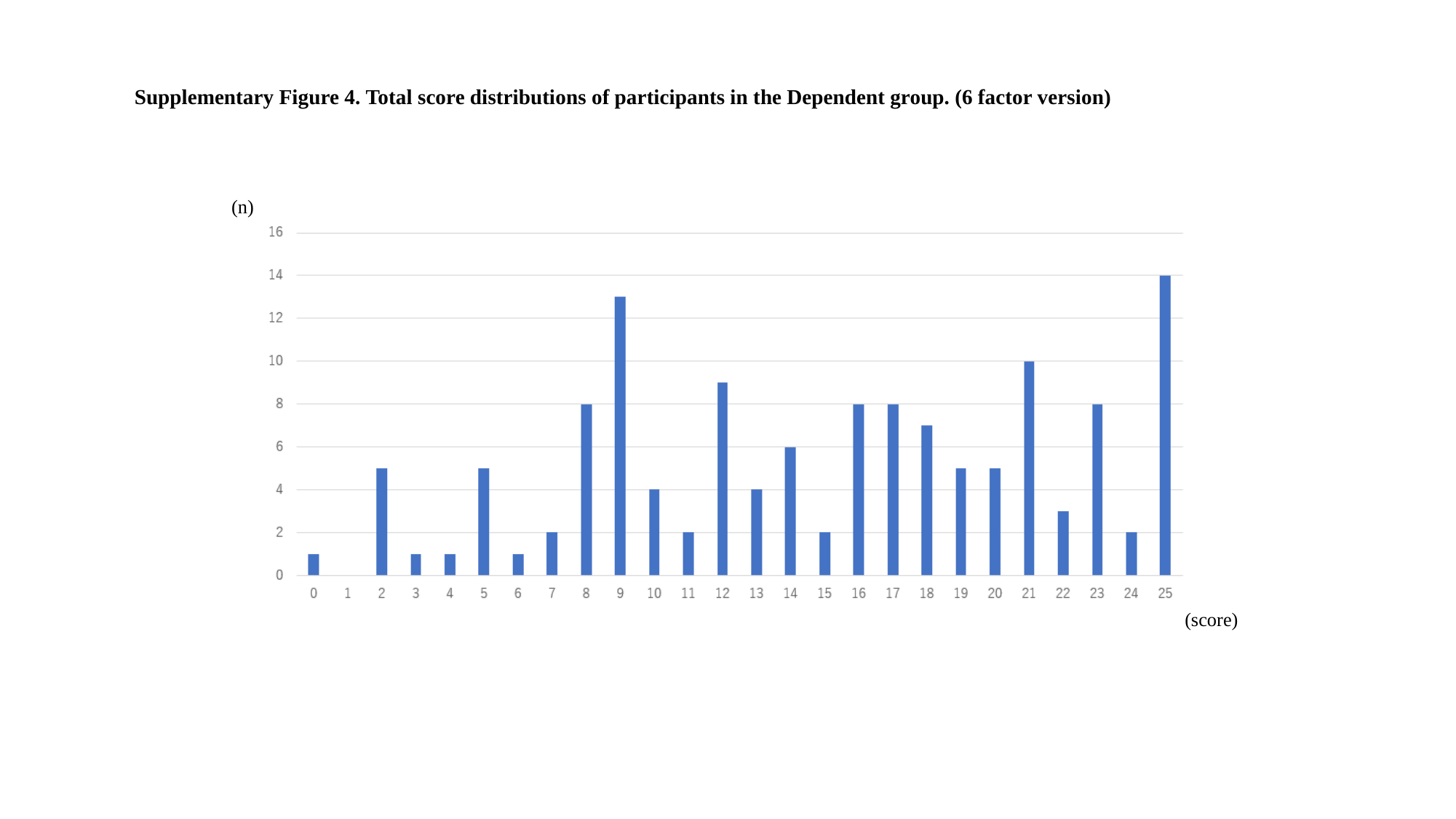

Supplementary Figure 4. Total score distributions of participants in the Dependent group. (6 factor version)
(n)
(score)
