# Supplementary material for: A scoring system and seven factors associated with certification for Japanese long-term care insurance in older people
Source: J Bone Miner Metab. 2025 May 28;43(4):419–29. doi: 10.1007/s00774-025-01606-x (PMC12279562; doi:10.1007/s00774-025-01606-x)
Supplement: Supplementary file 2 — Supplementary file2 (DOCX 66 KB) [file 774_2025_1606_MOESM2_ESM.docx]

**Supplementary Tables**

**Supplementary Table 1** Classification of nursing care need certification

| Levels | Condition |
| --- | --- |
| Assistance 1-2 | LTC is needed for some aspects of daily living, but proper care can improve or maintain ADL. |
| Long-term care level 1 | Unstable in rising and gait; partial support needed in toileting, bathing, etc. |
| Long-term care level 2 | Difficulty in rising and gait; partial or complete support needed in toileting, bathing, etc. |
| Long-term care level 3 | Impossible to rise and no gait. Complete support needed in toileting, bathing, dressing, and all other basic ADL. |
| Long-term care level 4 | Severe decline in ADL capacity; complete support needed in toileting, bathing, dressing, and all other basic ADL. |
| Long-term care level 5 | Bedridden state and complete support needed in all ADL; difficulty with communication. |

LTC: Long-Term Care

**Supplementary Table 2.** Factors analyzed

|  | Factors |  | Reference |
| --- | --- | --- | --- |
| 1 | Age |  | (1)(14) |
| 2 | Sex |  | (1)(2)(3) |
| 3 | Stroke |  | (1) |
| 4 | Heart failure |  | (1)(4) |
| 5 | Head injury |  | (5) |
| 6 | Parkinson’s disease |  | (1) |
| 7 | Cranial nerve-related diseases |  | (1)(5) |
| 8 | Hypertension |  | (1)(6) |
| 9 | Drinking |  | (6) |
| 10 | Smoking |  | (6) |
| 11 | Waight decrease |  | (7) |
| 12 | fatigue |  | (7) |
| 13 | Mood depression |  | (1) |
| 14 | Short exercise |  | (6)(7) |
| 15 | Regular exercise |  | (6)(7) |
| 16 | Heart attack |  | (1) |
| 17 | depression |  | (1)(9) |
| 18 | BMI |  | (6)(11)(14) |
| 19 | Diabetes |  | (1)(6)(12) |
| 20 | Exercise habits |  | (13) |
| 21 | Grip |  | (6)(8)(9)(10)(14)(15) |
| 22 | Walking speed |  | (6)(8)(9)(10)(14) |
| 23 | Albumin |  | (6)(16) |
| 24 | Dementia |  | (1)(6)(17) |
| 25 | Are you basically satisfied with your life? |  |  |
| 26 | Have you dropped many of your activities and interests? |  |  |
| 27 | Do you feel that your life is empty? |  |  |
| 28 | Do you often get bored? |  |  |
| 29 | Are you in good spirits most of the time? |  |  |
| 30 | Are you afraid that something bad is going to happen to you? |  |  |
| 31 | Do you feel happy most of the time? |  |  |
| 32 | Do you often feel helpless? | GDS15 | (6)(9)(18) |
| 33 | Do you prefer to stay at home, rather than going out and doing things? |  |  |
| 34 | Do you feel that you have more problems with memory than most? |  |  |
| 35 | Do you think it is wonderful to be alive now? |  |  |
| 36 | Do you feel worthless the way you are now? |  |  |
| 37 | Do you feel full of energy? |  |  |
| 38 | Do you feel that your situation is hopeless? |  |  |
| 39 | Do you think that most people are better off than you are? |  |  |
| 40 | Mobility |  |  |
| 41 | Self-care |  |  |
| 42 | Usual activities | EQ5D | (19) |
| 43 | Pain/Discomfort |  |  |
| 44 | Anxiety/Depression |  |  |
|  |  |  |  |
| 45 | Transfers  (Bed to chair and back) |  |  |
| 46 | Toilet use |  |  |
| 47 | Steirs |  |  |
| 48 | Feeding |  |  |
| 49 | Dressing | Barthel Index | (6)(20) |
| 50 | Bowels |  |  |
| 51 | Mobility  (on level surface) |  |  |
| 52 | Bladder |  |  |
| 53 | Bathing |  |  |
| 54 | Grooming |  |  |
|  |  |  |  |
| 55 | Can you use public transportation (bus or train) by yourself? |  |  |
| 56 | Are you able to shop for daily necessities? |  |  |
| 57 | Are you able to prepare meals by yourself? |  |  |
| 58 | Are you able to pay bills? |  |  |
| 59 | Can you handle your own banking? |  |  |
| 60 | Are you able to fill out forms for your pension? | IADL | (21)(22) |
| 61 | Do you read newspapers? |  |  |
| 62 | Do you read books or magazines? |  |  |
| 63 | Are you interested in news stories or programs dealing with health? |  |  |
| 64 | Do you visit the homes of friends? |  |  |
| 65 | Are you sometimes called on for advice? |  |  |
| 66 | Are you able to visit sick friends? |  |  |
| 67 | Do you sometimes initiate conversations with young people? |  |  |
|  |  |  |  |
| 68 | What is the year? |  |  |
| 69 | Season? |  |  |
| 70 | Month? |  |  |
| 71 | Day? |  |  |
| 72 | Date? |  |  |
|  | Where are we now? |  |  |
| 73 | State? |  |  |
| 74 | Country? |  |  |
| 75 | Town/city? |  |  |
| 76 | Hospital? |  |  |
| 77 | Floor? |  |  |
| 78  79  80 | What are the three objects?^*1^ |  |  |
| 81 | Count backward from 100 by sevens^*2^ | MMSE | (1)(6)(22) |
| 82  83  84 | What are the three objects?^*3^ |  |  |
| 85 | What is the two simple objects?^*4^ |  |  |
| 86 | Repeat the phrase: No ifs, ands, or buts. |  |  |
|  | The examiner gives the Patient  a piece of blank paper |  |  |
| 87 | “Take the paper in your right hand” |  |  |
| 88 | “Fold the paper in half” |  |  |
| 89 | “Put it on the floor” |  |  |
| 90 | Do what it says |  |  |
| 91 | Make up and write a sentence about anything |  |  |
| 92 | Copy the picture^*5^ |  |  |

*1 The examiner names three unrelated objects clearly and slowly, then the instructor asks the patient to name all three of them.

The patient’s response is used for scoring. The examiner repeats them until patient learn all of them, if possible.

*2 “I would like you to count backward from 100 by sevens.” (93, 86, 79, 72, 65, …)

*3 “Earlier I told you the names of three things. Can you tell me what those were?”

*4 Show the patient two simple objects, such as a wristwatch and a pencil, and ask the patient to name them.

*5 “Please copy this picture.” (The examiner gives the patient a blank piece of paper and asks him/her to draw the symbol below.

All 10 angles must be present and two must intersect.)

Reference:

1. Momose A, Yamaguchi S et al. (2021) Factors associated with long-term care certification in older adults:a cross-sectional study based on a nationally representive survey in Japan :BMC Geriatr Jun 21;374.

(2) Noguchi T, Kondo K et al. (2019) Community social capital and the onset of functional disability among older adults in Japan: A multilevel longitudinal study using Japan Gerontological Evaluation Study (JAGES) data: BMJ Open 9;e029279.

(3) Saito T, Murata C, et al. (2017) Cohort study on living arrangements of older men and women and risk for basic activities of daily living disability: Findings from the AGES project:BMC Geriatr 17;183.

(4) George A Heckman, Veronique M Boscart, et al. (2016) Managing Heart Failure in Long-term Care:Recommendations from an Interprofessional Stakeholder Consultation:Can J Aging Dec 35;447-464.

(5) M Doyinsola Bailey, Steven Gambert, et al. (2023) Traumatic Brain Injury and Risk of Long-Term Nursing Home Entry among Older Adults:An Analysis of Medicare Administrative Claims Data:J Neurotrauma Jan 40;86-93.

(6) Takahashi S, Yonekura Y, et al. (2022) Risk Factors of Long-Term Care Insurance Certification in Japan:A Scoping Review: Int J Environ. Res. Public Hearth 19;2162.

(7) Chen S, Honda T, et al. (2019) Physical frailty and ris

J Open 5; e008462.

(8) Shimada H, Makizako H, et al. (2015) Incidence of disability in frail older persons with or without slow k of needing long-term care in community-dwelling older adults: A 6-year prospective study in Japan: J. Nutr. Health. Aging 23;856–861.

(9) Tsutsumimoto K, Doi T, et al. (2016) Combined effect of slow gait speed and depressive symptoms on incident disability in older adults: J. Am. Med. Dir. Assoc17;123–127.

(10) Makizako H, Shimada H, et al. (2015) Impact of physical frailty on disability in community-dwelling older adults: A prospective cohort study: BMwalking speed: J. Am. Med. Dir. Assoc 16;690–696.

(11) Zhang S, Tomata Y, et al. (2019) The relationship between body mass index and disability-free survival in elderly Japanese: The Ohsaki Cohort 2006 Study: Int. J. Obes 43;2254–2263.

(12) Watanabe K, Okuro M, et al. (2018) Comorbidity of chronic kidney disease, diabetes and lower glycated hemoglobin predicts support/care-need certification in community-dwelling older adults: Geriatr. Gerontol. Int.18;521–529

(13) Hirai H, Kondo K, et al. (2009) Examination of risk factors for onset of certification of long-term care insurance in community-dwelling older people: AGES project 3-year follow-up study. Japanese Society of Public Health 56;501–512.

(14) Akune T, Muraki S. et al. (2014) Incidence of certified need of care in the long-term care insurance system and its risk factors in the elderly of Japanese population-based cohorts: The ROAD study. Geriatr. Gerontol. Int.14;695–701.

(15) Otsuka H, Kobayashi H, et al. (2020) Mobility performance among healthy older adults eligible for long-term care in Japan: A prospective observational study. Aging Clin. Exp. Res 32;1931–1937.

(16) Higashiguchi M, Nakaya N, et al. (2008) Malnutrition and the risk of long-term care insurance certification or mortality. A cohort study of the Tsurugaya Project: Japanese Society of Public Health 55;433–439.

(17) Tomata Y, Zhang S, et al. (2017) Changes in time spent walking and the risk of incident dementia in older Japanese people: The Ohsaki Cohort 2006 Study: Age Ageing 46;857–860.

(18) Shimada H, Lee S, et al. (2019) Prevalence of psychological frailty in Japan: NCGG-SGS as a Japanese National Cohort Study: J. Clin. Med.8;1554.

(19) Hata K, Nakabe T et al. (2022) Measuring the quality of life of long-term care service users in Japan:a cross-sectional questionnaire study: BMC Geriatrics 22;955.

(20) Makino K, Makizako H, et al. (2018) Impact of fear of falling and fall history on disability incidence among older adults: Prospective cohort study: Int. J. Geriatr. Psychiatry 33;658–662.

(21) Fujiwara Y, Amano H. et al (2006) Physical and psychological predictors for the onset of certification of long-term care insurance among older adults living independently in a community a 40-month follow-up study. Japanese Society of Public Health 53;77-91.

(22) Sagari A, Tabira T et al. (2021) Effect of dairy living Activities on the Need for Long-term Care in Older Adults: Asian J Occup Ther 17;69-77.

**Supplementary Table 3** Odds ratios of each EQ5D factor for LTC certification in participants

| Factors | Odds ratio (95% Cl) | P value | Odds ratio adjusted  by sex and age (95% Cl) | P value |
| --- | --- | --- | --- | --- |
| EQ5D score | 1.06e^-4^ (0.243e^-4^-4.59e^-4^) | 2.90e^-34^ | 0.0007 (1.52e^-4^-3.23e^-3^) | 1.22e^-20^ |
| Mobility | 17.8 (11.8-26.8) | 5.80e^-43^ | 8.77 (5.77-13.6) | 1.83e^-22^ |
| Self-care | 40.7 (21.7-76.3) | 6.26e^-31^ | 21.4 (10.7-42.9) | 6.19e^-18^ |
| Usual activities | 18.4 (12.3-27.4) | 2.31e^-46^ | 11.0 (7.13-16.8) | 9.79e^-28^ |
| Pain/Discomfort | 2.54 (1.90-3.40) | 3.39e^-10^ | 1.98 (1.41-2.78) | 8.17e^-5^ |
| Anxiety/Depression | 6.00 (3.91-9.22) | 2.50e^-16^ | 4.33 (2.58-7.25) | 2.59e^-8^ |

**Supplementary Table 4** Odds ratios of each Barthel Index factor for LTC certification in participants

| Factors | Odds ratio (95% Cl) | P value | Odds ratio adjusted  by sex and age (95% Cl) | P value |
| --- | --- | --- | --- | --- |
| Barthel Index score | 0.772 (0.734-0.812) | 5.22e^-24^ | 0.812 (0.768-0.857) | 8.08e^-14^ |
| Transfers  (Bed to chair and back) | 122 (28-528) | 1.51e^-10^ | 39.9 (7.87-202) | 8.60e^-6^ |
| Toilet use | 63.3 (18.3-219) | 5.52e^-11^ | 41.3 (10.1-169) | 2.35e^-7^ |
| Stairs | 54.5 (22.1-134) | 3.04e^-18^ | 19.1 (8.10-45.1) | 1.63e^-11^ |
| Feeding | 42.1 (15.5-115) | 2.69e^-13^ | 38.5 (11.8-126) | 2.69e^-13^ |
| Dressing | 38.5 (16.3-91.2) | 9.48e^-17^ | 21.0 (8.36-52.9) | 9.58e^-11^ |
| Bowels | 22.7 (10.5-49.1) | 2.00e^-15^ | 9.17 (4.30-19.6) | 1.00e^-8^ |
| Mobility  (on level surface) | 6.00 (3.90-9.22) | 3.03e^-16^ | 4.29 (2.96-6.23) | 1.61e^-14^ |
| Bladder | 5.57 (3.76-8.24) | 1.00e^-17^ | 3.68 (2.37-5.71) | 6.34e^-9^ |
| Bathing | 1.72e^8^ (0-Inf) | 0.960 | 4.49e^8^ (0-Inf) | 0.976 |
| Grooming | 6.23e^7^ (0-Inf) | 0.966 | 2.35e^8^ (0-Inf) | 0.975 |

**Supplementary Table 5** Odds ratios of each IADL factor for LTC certification in participants

| Factors | Odds ratio (95% Cl) | P value | Adjusted Odds ratio  by sex and age (95% Cl) | P value |
| --- | --- | --- | --- | --- |
| IADL score | 0.560 (0.514-0.609) | 6.07e^-41^ | 0.609 (1.26e^-6^-0.667) | 1.46e^-10^ |
| Can you use public transportation (bus or train) by yourself? | 0.016 (0.010-0.026) | 1.09e^-62^ | 0.027 (0.016-0.048) | 9.09e^-18^ |
| Are you able to shop for daily necessities? | 0.016 (0.009-0.029) | 1.55e^-47^ | 0.034 (0.018-0.065) | 9.93e^-25^ |
| Are you able to prepare meals by yourself? | 0.036 (0.023-0.056) | 8.15e^-49^ | 0.044 (0.024-0.080) | 5.02e^-25^ |
| Are you able to pay bills? | 0.019 (0.010-0.035) | 1.12e^-34^ | 0.040 (0.019-0.086) | 6.00e^-17^ |
| Can you handle your own banking? | 0.050 (0.032-0.077) | 1.61e^-39^ | 0.099 (0.058-0.170) | 3.48e^-17^ |
| Are you able to fill out forms for your pension? | 0.039 (0.025-0.061) | 2.13e^-45^ | 0.084 (0.050-0.141) | 9.59e^-21^ |
| Do you read newspapers? | 0.218 (0.146-0.326) | 1.14e^-13^ | 0.220 (0.134-0.362) | 2.54e^-9^ |
| Do you read books or magazines? | 0.230 (0.158-0.336) | 2.55e^-14^ | 0.299 (0.192-0.465) | 8.77e^-8^ |
| Are you interested in news stories or programs dealing with health? | 0.120 (0.075-0.193) | 1.85e^-18^ | 0.179 (0.097-0.332) | 4.71e^-8^ |
| Do you visit the homes of friends? | 0.128 (0.088-0.186) | 4.27e^-27^ | 0.206 (0.134-0.318) | 1.02e^-12^ |
| Are you sometimes called on for advice? | 0.118 (0.081-0.173) | 5.86e^-28^ | 0.238 (0.152-0.371) | 2.29e^-10^ |
| Are you able to visit sick friends? | 0.035 (0.021-0.057) | 1.33e^-41^ | 0.075 (0.042-0.135) | 4.35e^-18^ |
| Do you sometimes initiate conversations with young people? | 0.272 (0.179-0.414) | 1.18e^-9^ | 0.357 (0.215-0.593) | 6.85e^-5^ |

**Supplementary Table 6** Odds ratios of each GDS15 (Geriatric Depression Scale) factor for LTC certification in participants

| Factors | Odds ratio (95% Cl) | P value | Adjusted Odds ratio  by sex and age (95% Cl) | P value |
| --- | --- | --- | --- | --- |
| GDS score | 1.260 (1.190-1.340) | 1.58e^-14^ | 1.220 (1.140-1.310) | 3.26e^-8^ |
| Are you basically satisfied with your life? | 1.940 (1.230-3.080) | 4.65e^-3^ | 2.220 (1.290-3.820) | 4.08e^-3^ |
| Have you dropped many of your activities and interests? | 0.371 (0.256-0.539) | 1.88e^-7^ | 0.448 (0.291-0.689) | 2.60e^-4^ |
| Do you feel that your life is empty? | 0.299 (0.186-0.481) | 6.78e^-7^ | 0.307 (0.173-0.543) | 5.01e^-5^ |
| Do you often get bored? | 0.502 (0.337-0.748) | 7.03e^-4^ | 0.410 (0.258-0.653) | 1.69e^-4^ |
| Are you in good spirits most of the time? | 2.340 (1.220-4.480) | 0.01 | 1.940 (0.921-4.090) | 0.08 |
| Are you afraid that something bad is going to happen to you? | 1.240 (0.796-1.940) | 0.34 | 1.330 (0.805-2.190) | 0.27 |
| Do you feel happy most of the time? | 1.810 (0.799-4.120) | 0.15 | 1.790 (0.691-4.620) | 0.23 |
| Do you often feel helpless? | 0.195 (0.135-0.282) | 4.53e^-18^ | 0.246 (0.160-0.378) | 1.49e^-10^ |
| Do you prefer to stay at home, rather than going out and doing things? | 0.444 (0.309-0.638) | 1.13e^-5^ | 0.661 (0.435-1.000) | 5.27e^-2^ |
| Do you feel that you have more problems with memory than most? | 0.702 (0.491-1.010) | 5.33e^-2^ | 0.826 (0.550-1.240) | 0.36 |
| Do you think it is wonderful to be alive now? | 2.930 (1.750-4.910) | 4.27e^-5^ | 2.530 (1.370-4.680) | 3.04e^-3^ |
| Do you feel worthless the way you are now? | 0.207 (0.134-0.319) | 1.02e^-12^ | 0.419 (0.250-0.702) | 9.63e^-4^ |
| Do you feel full of energy? | 2.660 (1.850-3.820) | 1.37e^-7^ | 2.240 (1.480-3.400) | 1.50e^-4^ |
| Do you feel that your situation is hopeless? | 0.298 (0.187-0.477) | 4.42e^-7^ | 0.416 (0.239-0.725) | 1.99e^-3^ |
| Do you think that most people are better off than you are? | 0.837 (0.526-1.330) | 0.45 | 0.814 (0.481-1.380) | 0.44 |

**Supplementary Table 7** Odds ratios of each MMSE factor for LTC certification in participants

| Factors | Odds ratio (95% Cl) | P value | Odds ratio adjusted  by sex and age (95% Cl) | P value |
| --- | --- | --- | --- | --- |
| MMSE score | 0.764 (0.733-0.802) | 3.06e^-31^ | 0.832 (0.792-0.873) | 1.51e^-13^ |
| What is the year? | 0.151 (0.102-0.223) | 2.04e^-21^ | 0.287 (0.182-0.453) | 8.19e^-8^ |
| Season? | 0.100 (0.055-0.181) | 3.23e^-14^ | 0.171 (0.080-0.365) | 4.86e^-6^ |
| Month? | 0.033 (0.017-0.063) | 2.74e^-25^ | 0.058 (0.026-0.129) | 2.05e^-12^ |
| Day? | 0.077 (0.049-0.120) | 3.39e^-10^ | 0.141 (0.081-0.245) | 3.35e^-12^ |
| Date? | 0.052 (0.032-0.086) | 8.18e^-32^ | 0.100 (0.055-0.183) | 1.07e^-13^ |
| Where are we now? |  |  |  |  |
| State? | 0.011 (0.002-0.046) | 1.73e^-9^ | 0.030 (0.006-0.156) | 3.12e^-5^ |
| Country? | 0.021 (0.007-0.064) | 1.03e^-11^ | 0.092 (0.026-0.323) | 1.99e^-4^ |
| Town/city? | 0.224 (0.149-0.336) | 5.83e^-13^ | 0.482 (0.301-0.772) | 2.40e^-3^ |
| Hospital? | 0.058 (0.033-0.101) | 2.18e^-23^ | 0.094 (0.045-0.196) | 3.03e^-10^ |
| Floor? | 0.459 (0.312-0.675) | 7.43e^-5^ | 0.836 (0.533-1.310) | 0.437 |
| What are the three objects?^*1^ | 0.027 (0.007-0.098) | 4.86e^-8^ | 0.053 (0.011-0.262) | 3.16e^-4^ |
|  | 0.145 (0.078-0.272) | 1.71e^-9^ | 0.307 (0.143-0.661) | 2.55e^-3^ |
|  | 0.113 (0.050-0.254) | 1.32e^-7^ | 0.188 (0.069-0.511) | 1.06e^-3^ |
| Count backward from 100 by sevens^*2^ | 0.647 (0.584-0.716) | 7.05e^-17^ | 0.731 (0.650-0.822) | 1.17e^-7^ |
| What are the three objects?^*3^ | 0.173 (0.120-0.251) | 1.16e^-20^ | 0.387 (0.252-0.594) | 1.43e^-5^ |
|  | 0.206 (0.142-0.299) | 8.14e^-17^ | 0.372 (0.244-0.565) | 3.80e^-6^ |
|  | 0.177 (0.122-0.257) | 6.76e^-20^ | 0.405 (0.265-0.618) | 2.91e^-5^ |
| What are the two simple objects?^*4^ | 0.073 (0.018-0.293) | 2.27e^-4^ | 0.127 (0.037-0.431) | 9.48e^-4^ |
| Repeat the phrase: No ifs, ands, or buts. | 0.276 (0.180-0.423) | 3.58e^-9^ | 0.396 (0.236-0.663) | 4.35e^-4^ |
| The examiner gives the Patient  a piece of blank paper |  |  |  |  |
| “Take the paper in your right hand” | 0.488 (0.324-0.734) | 5.81e^-4^ | 0.825 (0.514-1.330) | 0.428 |
| “Fold the paper in half” | 0.334 (0.197-0.566) | 4.59e^-5^ | 0.530 (0.282-0.997) | 0.049 |
| “Put it on the floor” | 0.223 (0.136-0.368) | 3.62e^-9^ | 0.407 (0.224-0.740) | 3.24e^-3^ |
| Do what it says | 0.073 (0.031-0.172) | 2.40e^-9^ | 0.144 (0.052-0.402) | 2.13e^-4^ |
| Make up and write a sentence about anything | 0.161 (0.103-0.252) | 1.67e^-15^ | 0.242 (0.139-0.422) | 5.47e^-7^ |
| Copy the picture^*5^ | 0.143 (0.091-0.226) | 6.85e^-17^ | 0.272 (0.155-0.477) | 5.55e^-6^ |

*1 The examiner names three unrelated objects clearly and slowly, and then the instructor asks the patient to name all three of them.

The patient’s response is used for scoring. The examiner repeats them until patient learns all of them, if possible.

*2 “I would like you to count backward from 100 by sevens.” (93, 86, 79, 72, 65, …)

*3 “Earlier I told you the names of three things. Can you tell me what those were?”

*4 Show the patient two simple objects, such as a wristwatch and a pencil, and ask the patient to name them.

*5 “Please copy this picture.” (The examiner gives the patient a blank piece of paper and asks him/her to draw the symbol below.

All 10 angles must be present and two must intersect.)

**Supplementary Table 8** AUC (Area Under the ROC Curve) for each factor for Assistance or LTC certification

| Factors | AUC (95% CI) |
| --- | --- |
| Walking speed | 0.875 (0.842-0.907) |
| Age | 0.849 (0.814-0.884) |
| Grip | 0.811 (0.771-0.852) |
| Mobility (able to walk or bedridden) (EQ5D) | 0.805 (0.767-0.844) |
| Can you use public transportation (bus or train) by yourself? (IADL) | 0.794 (0.752-0.835) |
| Usual activities (EQ5D) | 0.769 (0.727-0.812) |
| Albumin levels | 0.737 (0.688-0.787) |
| Are you able to prepare meals by yourself? (IADL) | 0.732 (0.690-0.775) |
| Are you able to shop for daily necessities? (IADL) | 0.728 (0.685-0.770) |
| Dementia | 0.719 (0.672-0.765) |
| Are you able to fill out forms for your pension? (IADL) | 0.717 (0.674-0.759) |
| Do you visit the homes of friends? (IADL) | 0.715 (0.672-0.758) |
| Count backward from 100 by sevens^*1^ (MMSE) | 0.699 (0.652-0.746) |
| What are the three objects? (vehicle) second take (MMSE)^*2^ | 0.697 (0.655-0.740) |
| Are you sometimes called on for advice? (IADL) | 0.694 (0.650-0.737) |
| Are you able to visit sick friends? (IADL) | 0.693 (0.651-0.735) |
| Can you handle your own banking? (IADL) | 0.692 (0.650-0.734) |
| Self-care (EQ5D) | 0.690 (0.648-0.731) |
| What are three objects? (flower) second take (MMSE)^*2^ | 0.690 (0.647-0.734) |
| What are the three objects? (animal) second take (MMSE)^*2^ | 0.685 (0.643-0.728) |
| Regular exercise (At least once a week) | 0.681 (0.641-0.721) |
| Stair (Barthel Index) | 0.678 (0.637-0.718) |
| Are you able to pay bills? (IADL) | 0.670 (0.630-0.711) |
| Do you often feel helpless? (GDS) | 0.667 (0.623-0.711) |
| Mobility (on level surface. walking or wheelchair) | 0.658 (0.619-0.698) |
| What is the year? (MMSE) | 0.655 (0.613-0.698) |
| Day? (MMSE) | 0.655 (0.615-0.696) |
| Date? | 0.652 (0.612-0.692) |
| Dressing | 0.645 (0.606-0.684) |
| Pain/Discomfort | 0.637 (0.592-0.682) |
| Do you read books or magazines? | 0.634 (0.592-0.677) |
| Month? | 0.618 (0.581-0.654) |
| Do you read newspapers? | 0.616 (0.575-0.657) |
| Hypertension | 0.613 (0.575-0.652) |
| Bladder | 0.613 (0.576-0.651) |
| Town/city? | 0.610 (0.570-0.650) |
| Hospital? | 0.610 (0.574-0.647) |
| Do you feel full of energy? | 0.609 (0.565-0.653) |
| Anxiety/Depression | 0.608 (0.569-0.646) |
| Copy the picture^*3^ | 0.608 (0.570-0.646) |
| Are you interested in news stories or programs dealing with health? | 0.607 (0.570-0.645) |
| Make up and write a sentence about anything | 0.605 (0.567-0.643) |
| Short exercise (At least once a week) | 0.602 (0.550-0.653) |
| Toilet use (Barthel Index) | 0.601 (0.566-0.635) |
| Do you feel worthless the way you are now? (GDS) | 0.600 (0.562-0.639) |
| Have you dropped many of your activities and  interests? (GDS) | 0.599 (0.556-0.642) |
| Transfers (Bed to chair and back) (Barthel Index) | 0.593 (0.559-0.626) |
| Feeding (Barthel Index) | 0.592 (0.558-0.625) |
| Do you prefer to stay at home, rather than going out and doing things? (GDS) | 0.592 (0.548-0.636) |
| Do you sometimes initiate conversations with young people? (IADL) | 0.592 (0.553-0.631) |
| Repeat the phrase: No ifs, ands, or buds. (MMSE) | 0.587 (0.548-0.625) |
| Bowels (Barthel Index) | 0.586 (0.553-0.619) |
| Stroke | 0.585 (0.549-0.622) |
| Mood depression | 0.584 (0.536-0.633) |
| Fatigue | 0.579 (0.535-0.623) |
| Season? (MMSE) | 0.572 (0.541-0.604) |
| “Put it on the floor” | 0.569 (0.536-0.603) |
| Do you feel that your situation is hopeless? (GDS) | 0.567 (0.531-0.602) |
| Sex | 0.565 (0.525-0.605) |
| Do you feel that your life is empty? (GDS) | 0.564 (0.529-0.598) |
| Do you often get bored? (GDS) | 0.560 (0.520-0.600) |
| State? (MMSE) | 0.559 (0.531-0.587) |
| Country? (MMSE) | 0.558 (0.531-0.586) |
| What are three objects? (animal) first take (MMSE)^*2^ | 0.553 (0.525-0.582) |
| Do you think it is wonderful to be alive now? (GDS) | 0.549 (0.517-0.581) |
| “Fold the paper in half” (MMSE) | 0.548 (0.516-0.579) |
| Are you basically satisfied with your life? (GDS) | 0.542 (0.507-0.577) |
| Do what it says (MMSE) | 0.541 (0.517-0.566) |
| Cranial nerve-related diseases | 0.537 (0.512-0.561) |
| Heart failure | 0.536 (0.512-0.560) |
| What are three objects? (flower) first take (MMSE)^*2^ | 0.536 (0.513-0.560) |
| What are three objects? (vehicle) first take (MMSE)^*2^ | 0.536 (0.513-0.560) |
| Head injury | 0.532 (0.508-0.556) |
| Parkinson’s disease | 0.525 (0.505-0.544) |
| What are the two simple objects? (MMSE) ^*4^ | 0.525 (0.506-0.544) |
| Smoking | 0.464 (0.427-0.501) |
| Drinking | 0.454 (0.405-0.502) |

***1** “I would like you to count backward from 100 by sevens.” (93, 86, 79, 72, 65, …)

***2** The examiner names three unrelated objects clearly and slowly, and then the instructor asks the patient twice to name all three of them.

The patient’s response is used for scoring. The examiner repeats them until patient learns all of them, if possible.

The three objects are flower, animal and vehicle.

***3**“Please copy this picture.” (The examiner gives the patient a blank piece of paper and asks him/her to draw the symbol below.

All 10 angles must be present and two must intersect.)

***4** Show the patient two simple objects, such as a wristwatch and a pencil, and ask the patient to name them.

**Supplementary Table 9** AUC analysis on factors significantly associated with Dependency

| Factors | AUC (95% CI) |
| --- | --- |
| Walking speed | 0.875 (0.842-0.907) |
| Age | 0.896 (0.814-0.884) |
| Grip | 0.898 (0.771-0.852) |
| Mobility (able to walk or bedridden) (EQ5D) | 0.916 (0.767-0.844) |
| Can you use public transportation (bus or train) by yourself? (IADL) | 0.939 (0.752-0.835) |
| Usual activities (EQ5D) | 0.942 (0.727-0.812) |
| Albumin levels | 0.943 (0.688-0.787) |
| Are you able to prepare meals by yourself? (IADL) | 0.945 (0.690-0.775) |
| Are you able to shop for daily necessities? (IADL) | 0.945 (0.685-0.770) |

**Supplementary Table 10** Odds ratios of 6 factor for the Assistance or LTC certification from each reference value. (without albumin)

| Factors | Odds ratio (95% Cl) | P value |
| --- | --- | --- |
| Walking speed, m/s | 2.18 (1.85-2.57) | 1.52e^-20^ |
| (vs ≥1) |  |  |
| 0.8< <1 | 9.16 (5.37-15.6) | 4.34e^-16^ |
| ≤0.8 | 39.1 (21.0-72.8) | 8.08e^-31^ |
| Age, years | 1.24 (1.20-1.28) | 6.66e^-39^ |
| (vs 65-74) |  |  |
| 75-84 | 5.96 (3.34-10.7) | 1.65e^-9^ |
| 85-89 | 24.4 (12.6-47.2) | 2.15e^-21^ |
| 90- | 168 (69.9-404) | 2.25e^-30^ |
| Grip | 2.80 (2.33-3.35) | 7.62e^-29^ |
| (vs men≥28,  women≥18, kg) |  |  |
| Men<28, Women<18 | 8.48 (5.81-12.4) | 1.64e^-28^ |
| Mobility (EQ5D) | 17.8 (11.8-26.8) | 5.80e^-43^ |
| (vs able to walk) |  |  |
| bedridden | 18.3 (12.1-27.6) | 7.86e^-43^ |
| Able to use transportation by oneself (IADL) | 0.016 (0.01-0.03) | 1.09e^-62^ |
| (vs able) |  |  |
| Not able | 63.2 (38.9-103) | 1.09e^-62^ |
| Able to perform usual activities (EQ5D)^*1^ | 18.4 (12.3-27.4) | 2.31e^-46^ |
| (vs able) |  |  |
| Not able | 21.6 (14.4-32.4) |  |

LTC: Long-term care

*1 work, study, family and leisure activities.

**Supplementary Table 11** Scores associated with nursing care level. (without albumin)

| Predictors | Regression coefficient | score |
| --- | --- | --- |
| Walking speed, m/s |  |  |
| 0.8< <1 | 2.2151 | 3 |
| ≤0.8 | 3.6653 | 4 |
| Age, years |  |  |
| 75-84 | 1.7856 | 2 |
| 85-89 | 3.1957 | 4 |
| 90- | 5.1241 | 6 |
| Grip |  |  |
| Men<28, Women<18 | 2.1378 | 3 |
| Mobility (EQ5D) |  |  |
| bedridden | 2.9042 | 3 |
| Able to use transportation by oneself (IADL) |  |  |
| Not able | 4.1466 | 5 |
| Able to perform usual activities (EQ5D) |  |  |
| Not able | 3.0718 | 4 |

**Supplementary** **Table 12** Variance Inflation Factor (VIF) of the seven factors.

|  | Walking speed | Age | Grip | Mobility | Able to use public transportation by oneself | Able to perform usual activities |
| --- | --- | --- | --- | --- | --- | --- |
| Walking speed |  |  |  |  |  |  |
| Age | 1.1337 |  |  |  |  |  |
| Grip | 1.0499 | 1.1556 |  |  |  |  |
| Mobility | 1.1778 | 1.1560 | 1.0716 |  |  |  |
| Able to use public transportation by oneself | 1.2140 | 1.1160 | 1.0681 | 1.2167 |  |  |
| Able to perform usual activities | 1.1498 | 1.1046 | 1.0502 | 1.5977 | 1.3634 |  |
| Albumin levels | 1.0220 | 1.1001 | 1.0243 | 1.0543 | 1.0711 | 1.0527 |
